# Arsenic disrupts H3K9me3 and H3K27me3 balance by biasing PRC2.1 and PRC2.2 activity via PALI1 inhibition in carcinogenesis

**DOI:** 10.7150/ijbs.115605

**Published:** 2025-06-09

**Authors:** Haoyan Ji, Millie Elangbam, Yiran Qiu, Jessica Bamrah, Wenxuan Zhang, Aashna Pawar, Chitra Thakur, Fei Chen, Ziwei Wang

**Affiliations:** Stony Brook Cancer Center, Department of Pathology, Renaissance School of Medicine, Stony Brook University, Lauterbur Drive, Stony Brook, NY 11794, USA.

**Keywords:** arsenic, H3K9me3, H3K27me3, PALI1, PRC2, carcinogenesis

## Abstract

Inorganic arsenic (As^3+^) is a well-established human carcinogen, yet the molecular mechanisms underlying its oncogenic potential remain incompletely understood. Here, we show that exposure to environmentally relevant concentrations of As³⁺ disrupts chromatin architectures in human bronchial epithelial cells (BEAS-2B) by discordantly regulating two key repressive histone modifications: histone H3 lysine 27 trimethylation (H3K27me3) and H3K9me3. Chromatin immunoprecipitation and sequencing (ChIP-seq) reveals a genome-wide gain of H3K27me3 and a marked loss of H3K9me3 following As³⁺ treatment. Mechanistically, As^3+^ downregulates PALI1, an essential accessory subunit of the polycomb repressive complex 2.1 (PRC2.1), which uniquely coordinates H3K27me3 and H3K9me3 deposition via EZH2 and G9a, respectively. Loss of PALI1 impairs this dual repression mechanism, leading to widespread chromatin deregulation. Gene ontological analysis reveals that regions with diminished H3K9me3 in As³⁺-treated cells are enriched in pathways related to PRC2 activity, ribosomal biogenesis, stemness-associated transcription factors, xenobiotic metabolism (phases I and II), and GPCR signaling. Notably, these regions also include LINE-1 retrotransposons, whose de-repression is known to drive genomic instability—a hallmark of cancer. Given PALI1's potential tumor-suppressive role in lung, breast, and colon cancers, and other malignancies, its suppression by As³⁺ likely contributes to carcinogenesis through epigenetic reprogramming, genome destabilization, and activation of oncogenic transcriptional programs. These findings reveal a novel mechanism of As³⁺-induced epigenetic dysregulation and highlight the central role of histone modifications in environmental carcinogenesis.

## 1. Introduction

Chronic exposure to environmental arsenic, primarily through contaminated drinking water, food, and soil, represents a major global health concern, particularly in regions with naturally high arsenic levels or significant industrial pollution 1. Trivalent arsenic (As³⁺) is linked to a spectrum of adverse health effects, including dermatological conditions, cardiovascular diseases, neurotoxicity, and diabetes 2. Extensive epidemiological studies have established its carcinogenic potential, leading to its classification as a Group 1 carcinogen by the International Agency for Research on Cancer (IARC) 3, 4. Populations consuming arsenic-contaminated water face an elevated risk of skin cancer, particularly in highly affected areas such as Bangladesh, Taiwan, and Chile, where arsenic concentrations surpass the World Health Organization's safety threshold of 10 ppb 5. In addition to skin cancer, arsenic exposure is strongly correlated with increased incidences of lung, bladder, liver, breast, kidney, and prostate cancers, with cohort studies confirming heightened cancer mortality rates in exposed populations 6-10.

As^3+^-induced carcinogenesis involves multiple interconnected biological pathways. In addition to the generation of reactive oxygen species (ROS), which cause DNA damage, lipid peroxidation, and protein oxidation, As^3+^ binds directly to DNA and proteins, forming crosslinks that impair DNA repair pathways such as base excision repair (BER) and nucleotide excision repair (NER), thereby increasing mutation rates 11, 12. Moreover, As^3+^ activates several oncogenic signaling pathways, including MAPK/ERK, Nrf2, STAT3, PI3K/AKT, NF-κB, and Wnt/β-catenin, promoting cell proliferation, survival, and tumorigenesis 13-17. Additionally, As^3+^ induces genomic instability through chromosomal aberrations, telomere dysfunction, and micronucleus formation, while also disrupting cell cycle by altering cyclins, cyclin-dependent kinases (CDKs), and tumor suppressors 18-20. Chronic As^3+^ exposure triggers inflammation by activating pro-inflammatory cytokines and immune cell recruitment while suppressing immune surveillance 21. Furthermore, As^3+^ enhances angiogenesis and metastasis by upregulating vascular endothelial growth factor (VEGF) and increasing matrix metalloproteinase (MMP) activity 22. Collectively, these mechanisms drive malignant transformation and the emergence of cancer stem-like cells (CSCs), emphasizing the need to understand these pathways for the development of targeted therapies and preventive strategies.

Despite significant progress in elucidating arsenic's carcinogenic mechanisms, its impact on the epigenetic landscape remains poorly understood. Among the most critical epigenetic modifications is the methylation of arginine (R) and lysine (K) residues in histone N-terminal tails, particularly histone H3 lysine 4 trimethylation (H3K4me3), H3K9me3, and H3K27me3, which play key roles in chromatin regulation 23. In general, H3K4me3 is associated with transcriptionally active chromatin, whereas H3K9me3 and H3K27me3 are considered as repressive markers that facilitate heterochromatin formation and gene silencing 24. We previously demonstrated that As^3+^ significantly alters histone methylation. In As^3+^-transformed bronchial epithelial cells, long-term exposure to environmentally relevant concentrations of As^3+^ led to an increase in H3K4me3 levels across the genome, except for chromosome Y, while modestly decreasing H3K27me3 25. However, whether these epigenetic changes were directly caused by As^3+^ or a consequence of malignant transformation remained unclear. To address this question, in the present study, we treated human bronchial epithelial cell line BEAS-2B with 1 μM As^3+^ for 6 h and observed a discordant effect on repressive histone markers: As³⁺ downregulated H3K9me3 while upregulating H3K27me3. Further investigation revealed that this imbalance was mediated by a selective shift between two variants of the polycomb repressive complex 2 (PRC2), PRC2.1 and PRC2.2. Both PRC2.1 and PRC2.2 share EZH2, the core methyltransferase responsible for H3K27me3 deposition 26. However, PRC2.1 uniquely contains PALI1 (PRC2-associated LCOR isoform 1), an auxiliary protein that recruits and interacts with G9a, a methyltransferase responsible for H3K9me1, H3K9me2 and H3K9me3 27. This interaction allows PRC2.1 to regulate both H3K27me3 and H3K9me3. Our findings indicate that As^3+^ represses PALI1 expression, thereby shifting the balance from PRC2.1 to PRC2.2. This selective inhibition of PRC2.1 weakens H3K9me3 deposition, which is essential for maintaining genomic stability. Given the critical role of H3K9me3 in genome integrity, including repression of transposable elements in the genome 28, PALI1 suppression by As³⁺ may contribute to carcinogenesis by destabilizing heterochromatin, de-repressing transposable elements, and promoting epigenetic dysregulation. Collectively, these findings reveal a novel mechanism by which As³⁺ perturbs the histone methylation landscape, providing new insights into its role in epigenetic reprogramming during carcinogenesis. Understanding how As³⁺ selectively biases PRC2 variants could inform therapeutic strategies targeting histone methylation dysregulation in As³⁺-related cancers.

## 2. Materials and Methods

### 2.1. Cell culture

The human bronchial epithelial cell line BEAS-2B was purchased from ATCC (Manassas, VA). BEAS-2B cells were cultured in Dulbecco's Modified Eagle's Medium (DMEM) (Sigma-Aldrich, St. Louis, MO), supplemented with 5% (v/v) fetal bovine serum (R&D system, Minneapolis, MN), 1% (v/v) L-glutamine, and 1% (v/v) penicillin-streptomycin at 37°C in a humidified incubator with 5% CO₂.

### 2.2. Chromatin Immunoprecipitation Sequencing Analysis (ChIP-seq)

A total of 1.0 ×10^6^ BEAS-2B cells were seeded in a 10 cm dish and treated with 1 µM As^3+^ for 6 h. When cells reached 90% confluence, they were fixed with formaldehyde solution, quenched with glycine, washed with PBS, and snap-frozen on dry ice 29. Chromatin was sheared and immunoprecipitated using ChIP-grade antibodies against Nrf2, AHR, H3K4me3, H3K9me3, and H3K27me3 (Active Motif, Carlsbad, CA, USA). Input and control DNA were prepared, followed by sequencing and data analysis as described previously 30.

### 2.3. RNA sequencing (RNA-seq)

BEAS-2B cells (1 ×10^6^) were treated with 2 µM As^3+^ for 12 h. Total RNA was extracted, and RNA quality was assessed using an Agilent Bioanalyzer. Genome hg19 was used as a reference. RNA-Seq data analysis was performed as previously described 31.

### 2.4. Western blot analysis and Co-immunoprecipitation (Co-IP)

Western blot and Co-IP were conducted as described previously 32. Briefly, cells were lysed in RIPA buffer (Cell Signaling, Danvers, MA) supplemented with a protease/phosphatase inhibitor cocktail and 1 mM PMSF (Thermo Fisher Scientific, Waltham, MA). Lysates were sonicated, centrifuged at 4°C, and the supernatant was collected. Protein concentration was then measured using a BCA Protein Assay Reagent Kit (Thermo Fisher Scientific). Samples were prepared in 4 × LDS sample buffers, denatured at 98°C for 10 min, resolved by SDS-PAGE, and transferred onto PVDF membranes (MilliporeSigma, Burlington, MA). Membranes were blocked with BSA for 1 h at room temperature, incubated with primary antibodies overnight at 4°C, and then with secondary antibodies for 2 h at room temperature. Signals were detected using ECL reagent (Thermo Fisher Scientific) and visualized with a ChemiDoc Imaging System (Bio-Rad, Hercules, CA). For co-IP, cell lysates were incubated overnight at 4°C with primary antibodies on a rocker. Pre-washed Protein A/G magnetic beads were then added and incubated for 1 h at room temperature. Beads were washed with IP wash buffer and ultrapure water before proteins were eluted via low-pH elution or high-temperature boiling. Protein complexes were resolved by SDS-PAGE for further analysis.

### 2.5. Real-time PCR and Regular PCR

Total RNA was extracted using the RNeasy Plus Mini Kit (Qiagen, Germantown, MD), quantified, and reverse transcribed into cDNA using the High-Capacity cDNA Reverse Transcription Kit (Applied Biosystems, Waltham, MA). Real-time PCR was performed using SYBR Green PCR Master Mix (Applied Biosystems) on a Roche LightCycler 480 instrument. For conventional PCR, cDNA was synthesized using SuperScript™ IV Reverse Transcriptase (Thermo Fisher Scientific) and amplified with DreamTaq™ Green PCR Master Mix (Thermo Fisher Scientific). PCR products were analyzed by nucleic acid gel electrophoresis and visualized using a ChemiDoc Imaging System (Bio-Rad).

### 2.6. CRISPR-Cas9 gene editing

CRISPR single-guide RNAs (sgRNAs) targeting AHR and Nrf2 were designed using the CRISPR Design Tool (http://crispr.mit.edu/), as described previously 31, 33. The sgRNA sequences were:

- AHR: 5'-AGCGGCATAGAGACCGACTT-3' (targeting exon 1 and exon 2)
- Nrf2: 5'-AGCGGCATAGAGACCGACTT-3' (targeting exon 2)

Sense and antisense primers for each sgRNA were annealed at 95°C for 5 min, then cooled to 25°C at a rate of 5°C/min. The pSpCas9-2A-Blast vector (Addgene, Watertown, MA) was linearized with Bpil and dephosphorylated using FastAP (Thermo Fisher Scientific) at 37°C for 30 min. sgRNA pairs were ligated into the linearized vector using T4 DNA ligase (Thermo Fisher Scientific) at 22°C for 10 min. Ligation products were transformed into DH5α competent *E. coli* (Thermo Fisher Scientific) following the manufacturer's protocol.

### 2.7. Statistics

ChIP-seq and RNA-seq data were subjected to quality control by Active Motif (Carlsbad, CA). Statistical analyses were conducted using GraphPad Prism 7 (GraphPad Software, San Diego, CA). Unless otherwise specified, all experiments were performed independently in triplicate, with a minimum of three biological replicates. Differences between groups were analyzed using Student's *t*-test or one-way ANOVA. A *p*-value < 0.05 was considered statistically significant, and all tests were two-sided.

## 3. Results

### 3.1. As^3+^ decreases H3K9me3 while increases H3K27me3 levels across the genome

**Figure 1:**
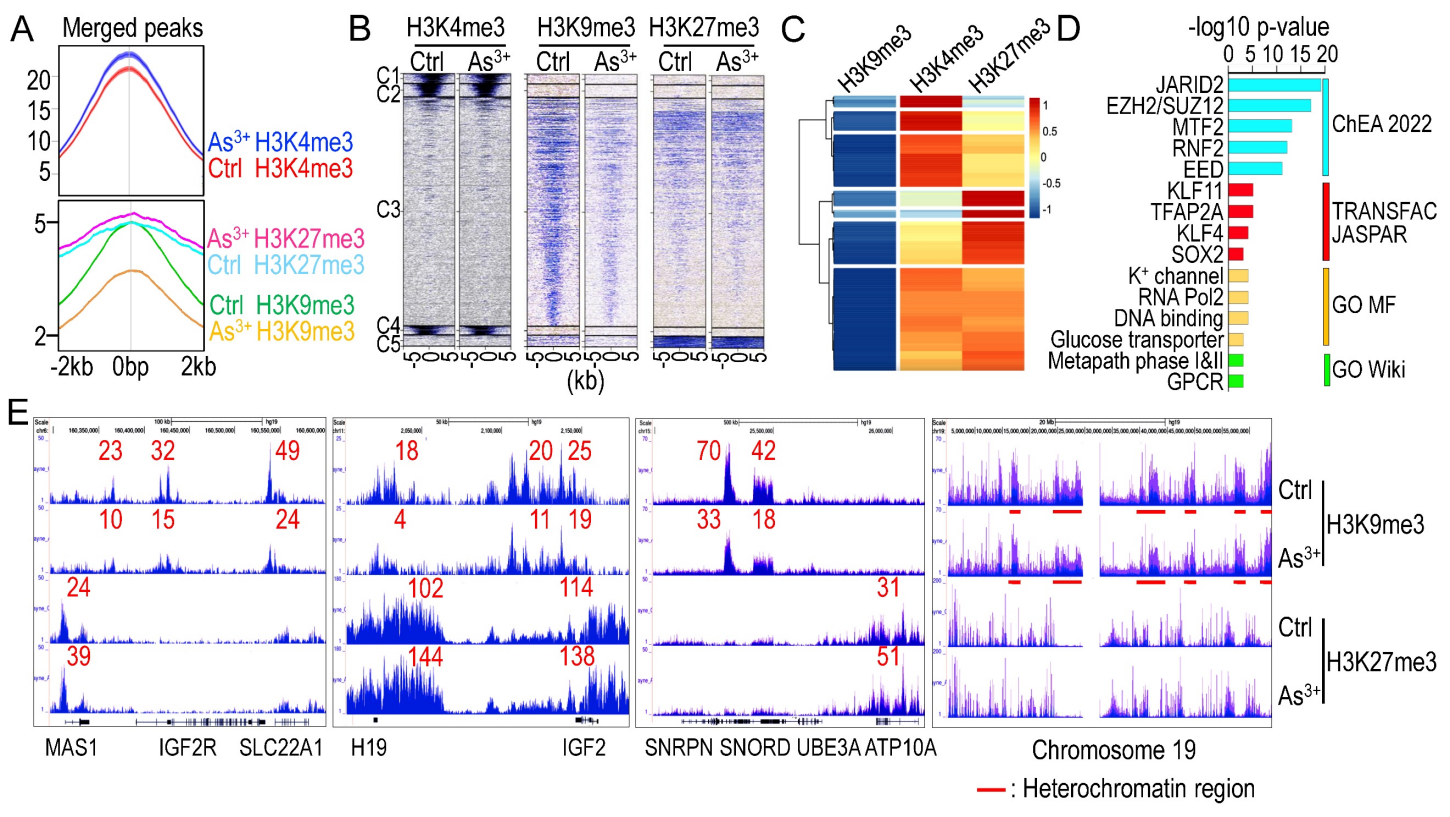
**Opposite regulation of As^3+^ on H3K9me3 and H3K27me3. A** Average ChIP-seq merged peak signals of H3K4me3 (top), H3K9me3, and H3K27me3 (bottom) in control cells and cells treated with 1 μM As^3+^ for 6 h. **B** Heatmaps of merged peaks for H3K4me3, H3K9me3, and H3K27me3. **C** Clustering analysis of 1,405 protein-coding genes exhibiting As³⁺-induced H3K9me3 downregulation (log₂FC < -0.5). Heatmap shows the relative enrichment levels of H3K9me3, H3K4me3, and H3K27me3 across these genes. Most genes displayed increased H3K4me3 enrichment, indicative of transcriptional activation, while a subset showed concurrent elevation of H3K27me3, suggesting complex epigenetic reprogramming. Gene enrichment scores are shown as log2 fold changes. **D** Pathway analysis of these 1,405 genes with reduced H3K9me3 in As^3+^-treated cells show significant enrichment of PRC2 components, stemness transcription factors, K^+^ channel, transporter, and G-protein-coupled receptor (GPCR). **E** Enrichment levels of H3K9me3 and H3K27me3 in ChIP-seq for the indicated imprint genes and heterochromatin regions of chromosome 19.

To investigate whether transient As^3+^ treatment alters overall histone methylation, we treated BEAS-2B cells with 1 μM As^3+^ for 6 h, followed by ChIP-seq analysis, focusing on two well-characterized repressive lysine methylation markers: H3K9me3 and H3K27me3. These markers often correlate across the genome or within individual genes in response to extracellular signals 34. Intriguingly, short-term As^3+^ treatment led to a global increase in H3K4me3 and H3K27me3 levels but a significant decrease in H3K9me3 levels (**Figs. 1A & 1B**). A total of 1,405 protein-coding genes exhibited As^3+^-induced H3K9me3 downregulation, with a Log2 fold change (FC) of less than -0.5. Gene clustering analysis of these genes revealed that the majority showed increased enrichment of H3K4me3, a promoter-associated marker of active transcription, with a subset also displaying elevated H3K27me3 levels (**Fig. 1C**). Gene ontology analysis indicated that many of these genes are regulated by the PRC2 complex (JARID2, EZH2/SUZ12, MTF2, EED) and stemness transcription factors such as KLF4 and SOX2. Functional pathway analysis suggests that many of these genes are related to potassium (K^+^) channels, RNA transcription, metabolism, and GPCR signaling (**Fig. 1D**). Examination of individual gene loci showed that most imprinted genes, including IGF2R, H19, IGF2, SNORD, and ATP10A, exhibited decreased H3K9me3 enrichment but elevated H3K27me3 levels, a pattern also observed in the majority of heterochromatin regions (**Fig. 1E** and data not shown).

### 3.2. Loss of H3K9me3 enrichment on long interspersed nuclear elements-1 (LINE-1, L1)

**Figure 2:**
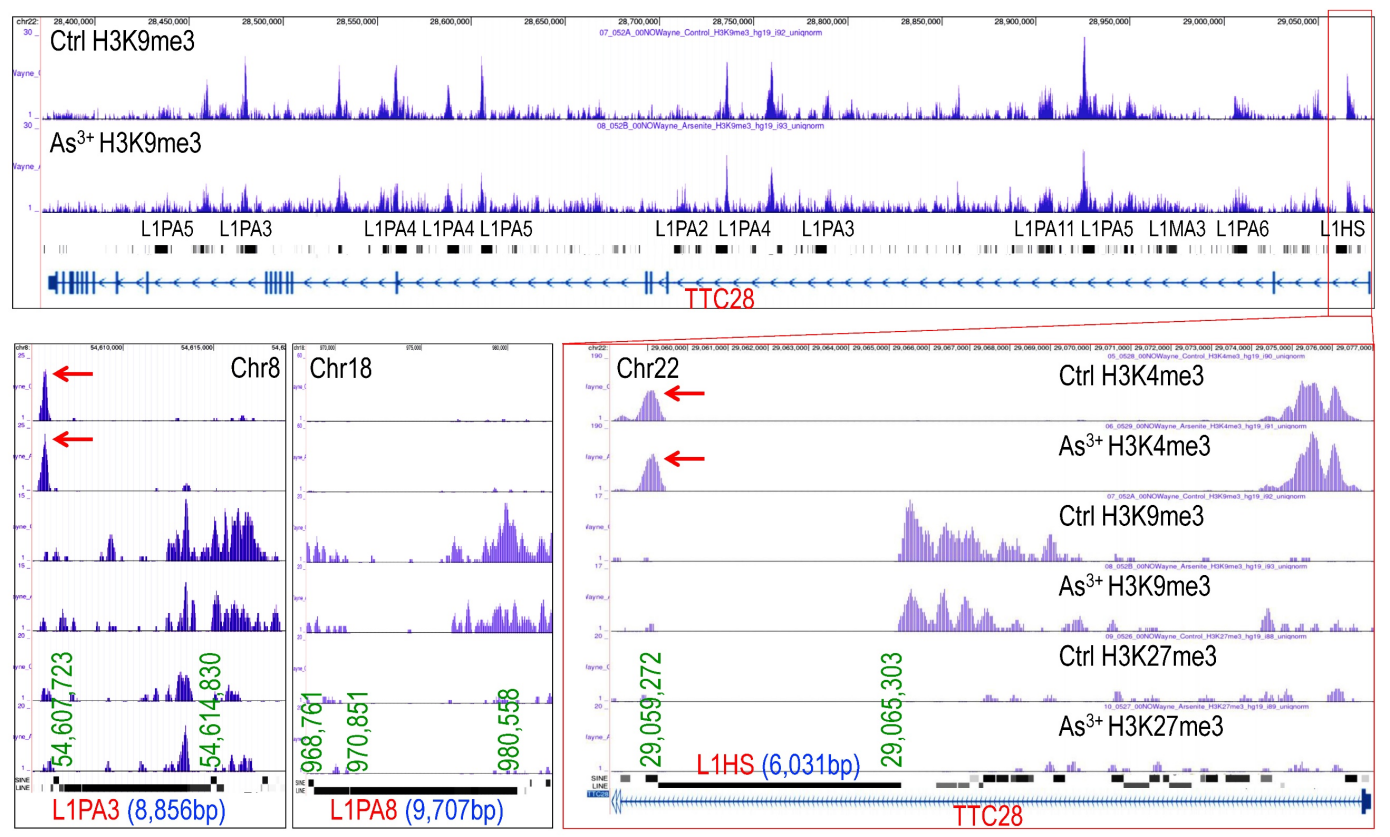
**As^3+^ reduces H3K9me3 levels on the indicated LINE-1 elements.** Genome browser screenshots show decreased H3K9me3 and increased H3K4me3 at selected LINE-1 elements, including hominoid-specific L1PA3 on chromosome 8 (Chr8), LIPA8 on chromosome 18 (Chr18) and human-specific L1HS in the first intron of *TTC28* on chromosome 22 (Chr22), in As³⁺-treated cells. These histone modifications suggest a transcriptionally permissive state at these retrotransposon loci.

LINE-1 (L1) elements are autonomous retrotransposons making up ~17% of the human genome. While most are evolutionarily ancient and inactive, a small subset of active and young LINE-1 elements remains capable of mobilization via a reverse transcription “copy-and-paste” mechanism. Active LINE-1s encode two essential proteins, ORF1p and ORF2p, for retrotransposition. Normally silenced epigenetically, LINE-1 can become reactivated in diseases like cancer, contributing to genomic instability and abnormal gene expression 28. ChIP-seq analysis revealed that both evolutionarily ancient and young LINE-1 elements featured with a marked loss of H3K9me3 without a corresponding gain in H3K27me3 in As³⁺-exposed cells, as exampled in **Fig. 2**, indicating failure of compensatory repression. Notably, specific LINE-1 subfamilies, including hominoid-specific L1PAs and human-specific L1HS elements, such as L1PA3 on chromosome 8 (Chr8) and L1HS located in the first intron of the TTC28 gene on chromosome 22 (Chr22), exhibited prominent H3K4me3 peaks, a hallmark of active transcription (**Fig. 2**). These findings suggest that As³⁺-induced H3K9me3 depletion at LINE-1 loci may establish a transcriptionally permissive or poised chromatin state, promoting retrotransposon activation, genomic instability, and the acquisition of stem-like and oncogenic features in exposed cells.

### 3.3. As^3+^ impairs PRC2.1 activity by repressing PALI1 expression

**Figure 3:**
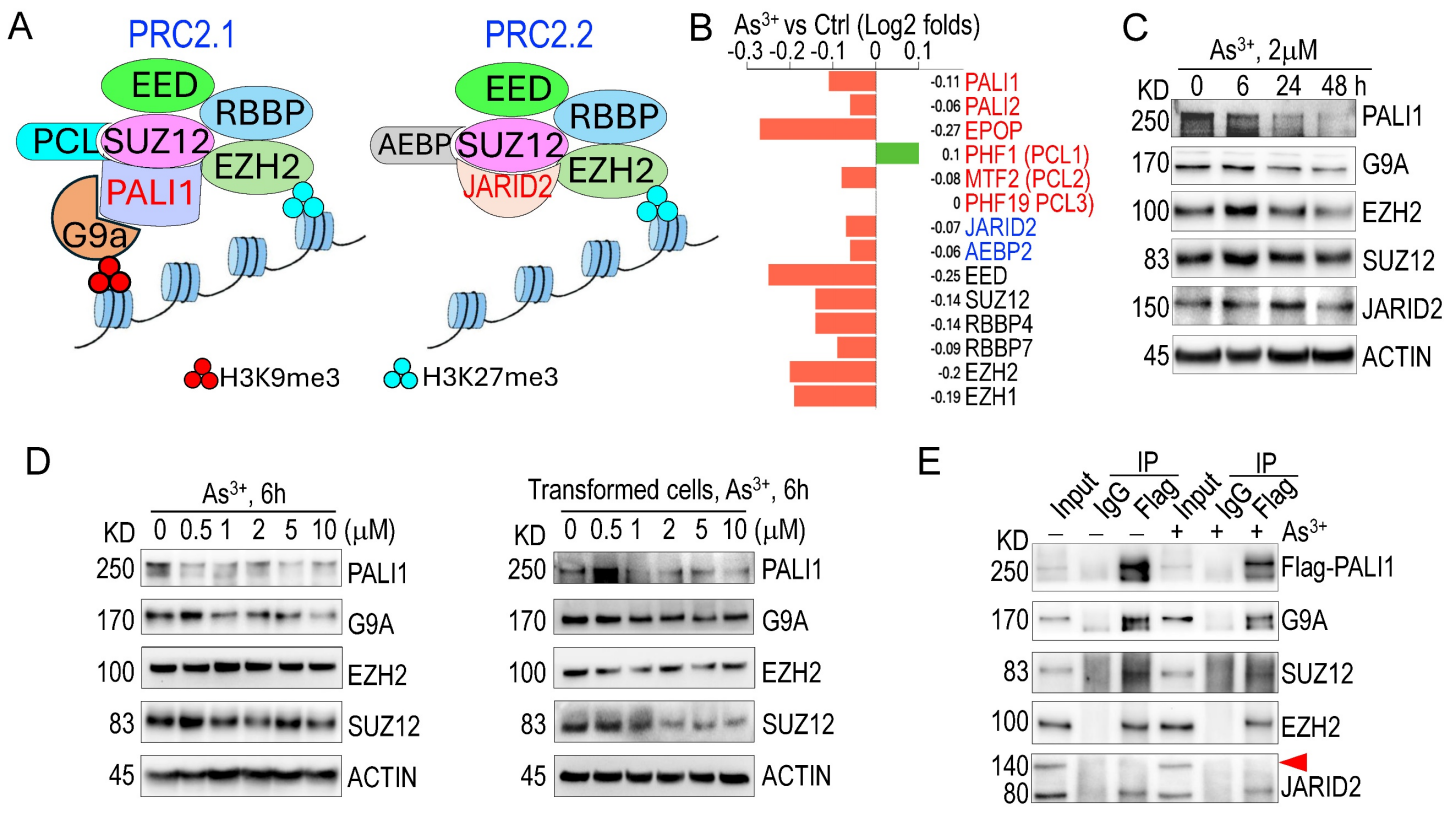
** As^3+^ compromises PRC2.1 activity by inhibiting PALI1. A** The diagram highlights the similarities and differences in subunits, auxiliary proteins, and functions between PRC2.1 and PRC2.2. **B** RNA-seq analysis shows the gene expression of key PRC2 subunits in response to As^3+^ treatment. Gene expression results are shown as log2 fold changes. **C** Western blot analysis demonstrates the time-dependent effects of As^3+^ on the indicated proteins. **D** Western blot analysis showing the dose-dependent effects of As³⁺ (0.5-10 μM, 6 h) on the indicated proteins in parental BEAS-2B cells (left) and As³⁺-transformed BEAS-2B cells (right). Protein expression patterns were compared to assess acute responsiveness and sustained alterations following chronic As^3+^ exposure. **E** Co-IP assays reveal an interaction between Flag-PALI1 with G9a, SUZ12, EZH2, but not with full-length JARID2 (indicated by the red-filled triangle).

Given that genes exhibiting significant H3K9me3 reduction in response to As^3+^ are predominantly regulated by PRC2 (**Fig. 1D**), and considering PRC2's crucial role in catalyzing both H3K9me3 and H3K27me3 35, we investigated whether As^3+^ affects the expression of PRC2 subunits. PRC2 exists in two major subcomplexes: PRC2.1 and PRC2.2 36, 37. PRC2.1 catalyzes H3K27me3 via the core subunit EZH2 and H3K9me3 through the auxiliary protein PALI1, which recruits and interacts with the H3K9-specific methyltransferase G9a (EHMT2) 35. In contrast, PRC2.2 incorporates JARID2 as an auxiliary subunit but lacks the ability to bind G9a and is therefore limited to H3K27me3 activity (**Fig. 3A**). RNA-seq analysis revealed that 12-h exposure to 2 µM As³⁺ broadly downregulates transcripts encoding key PRC2 components, including *PALI1*, *PALI2*, *EPOP*, *MTF2*, *JARID2*, *AEBP2*, *EED*, *SUZ12*, *RBBP4*, *RBBP7*, *EZH2*, and *EZH1* (**Fig. 3B**). Western blotting further confirmed that As^3+^ exposure leads to a marked downregulation of the PALI1 protein in both time- and dose-dependent manners (**Figs. 3C & 3D**). A modest reduction in G9a protein levels was also observed; however, the protein levels of EZH2, SUZ12, and JARID2 remained largely unchanged following acute As³⁺ treatment. To assess whether these effects persist under chronic arsenic exposure, a more physiologically relevant condition for malignant transformation, we examined the expression of PALI1 and its associated epigenetic regulators in a malignantly transformed BEAS-2B cell model. This model was established through continuous exposure to 0.25 µM As³⁺ for three months. The transformed cells were then treated with 0.5-10 µM As³⁺ for an additional 6 h to evaluate dose responsiveness. Consistent with our acute exposure data, PALI1 and G9a were significantly repressed in a dose-dependent manner in these transformed cells. SUZ12 also showed a dose-dependent decrease, while EZH2 levels remained relatively stable and did not exhibit a consistent dose-response trend (**Fig. 3D**). These results suggest that PALI1 downregulation is a sustained consequence of chronic arsenic exposure and may contribute to the maintenance of a pro-oncogenic epigenetic state. To determine whether As^3+^ treatment disrupts the assembly of the PRC2.1 complex, we overexpressed Flag-tagged PALI1 and conducted co-immunoprecipitation (co-IP) in cells treated with 2 μM As^3+^ for 6 h. Despite the notable reduction in Flag-PALI1 protein levels in As^3+^-treated cells, its interaction with G9a, SUZ12, and EZH2 remained intact (**Fig. 3E**). As expected, no interaction between PALI1 and full-length JARID2 was detected in either control or As^3+^-treated cells, supporting the notion that PALI1 and JARID2 are components of mutually exclusive PRC2 subcomplexes. Interestingly, PALI1 was found to interact with a truncated 80 kDa isoform of JARID2 (**Fig. 3E, the JARID2 panel**), previously identified as ΔN-JARID2 38. The functional relevance of this interaction remains to be elucidated.

### 3.4. As^3+^ disrupts PALI1 mRNA splicing

**Figure 4:**
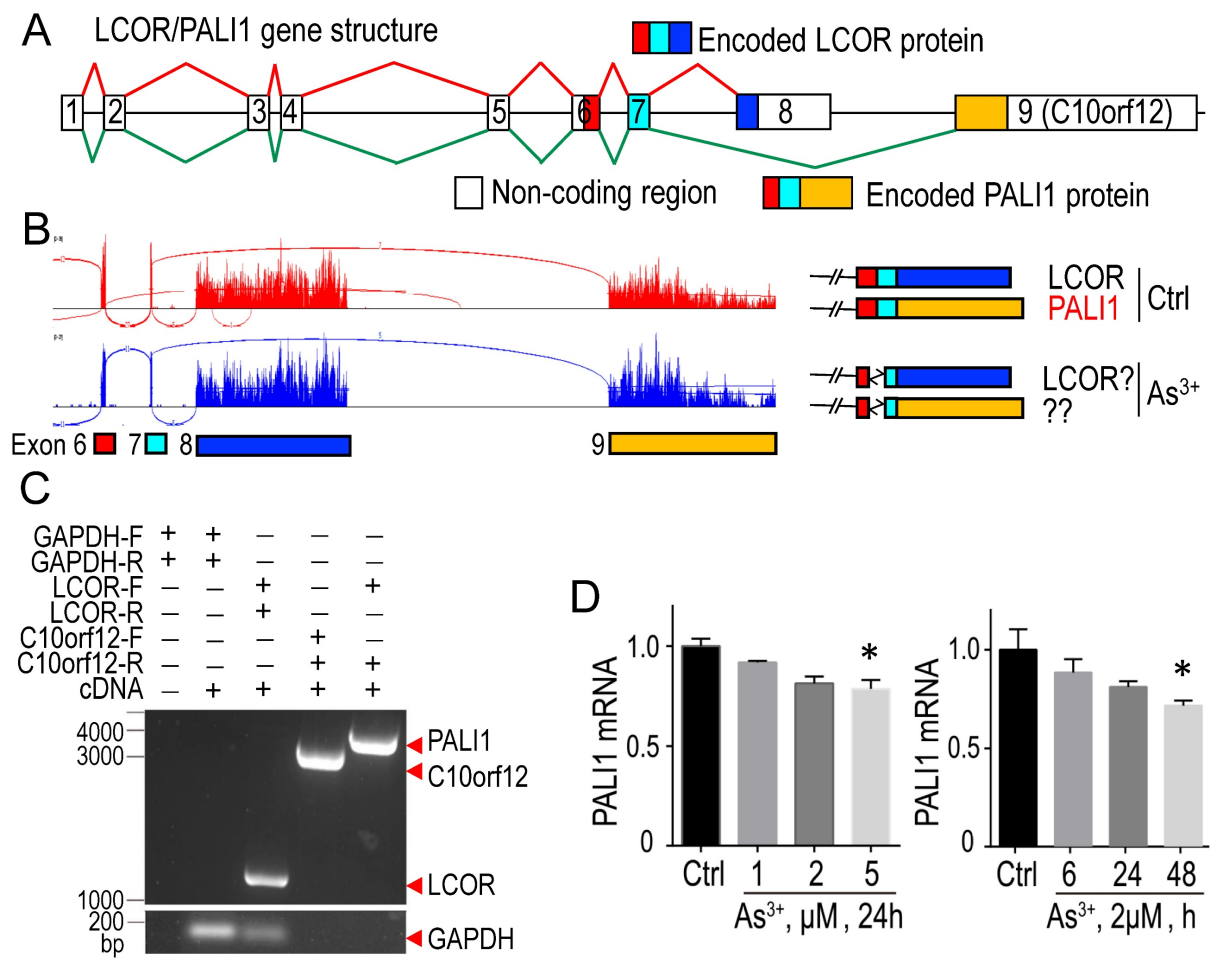
** Treatment with As^3+^ interferes with the splicing of PALI1 mRNA. A** The diagram illustrates the splicing of LCOR mRNA and its alternative splicing to produce PALI1 mRNA. **B** RNA-seq spectra reveals that As^3+^ induces the selection of alternative donor site and acceptor splice sites between exons 6 and 7. **C** PCR amplification of PALI1, C10orf12, and LCOR, with GAPDH as an internal control, in BEAS-2B cells. **D** qPCR demonstrates that As^3+^ treatment moderately suppresses PALI1 mRNA expression in a dose-dependent manner (left) and a time-dependent manner (right). Data are expressed as mean ± SD, n = 6, *p <0.05 vs. control (Ctrl).

PALI1 is generated via alternative splicing of LCOR mRNA, involving exon 8 skipping and exon 9 (C10orf12) inclusion (**Fig. 4A**) 39. In control cells, RNA-seq indicated equal splicing to produce both LCOR and PALI1 transcripts. In As^3+^-treated cells, alternative splicing donor and acceptor sites were used between exons 6 and 7, potentially disrupting PALI1 formation (**Fig. 4B**). Since exon 9 was previously defined as an independent transcript, C10orf12, located near the LCOR gene, it is important to determine whether PALI1 transcripts are truly produced in these cells 40. To address this, we designed PCR primers to detect the transcripts of LCOR, C10orf12, and PALI1. PCR results confirmed the presence of LCOR, C10orf12, and PALI1 transcripts (**Fig. 4C**). Quantitative PCR (qPCR) analysis showed that As^3+^ specifically inhibits PALI1 mRNA generation, particularly at 5 μM As^3+^ for 24 h, or 2 μM As^3+^ for 48 h (**Fig. 4D**). These findings suggest that, unlike other PRC2 subunits, PALI1 is uniquely susceptible to As^3+^-induced inhibition at both protein and mRNA levels, leading to functional impairment of PRC2.1.

### 3.5. Reciprocal regulation of Nrf2 and AHR in As^3+^-induced PALI1 inhibition

**Figure 5:**
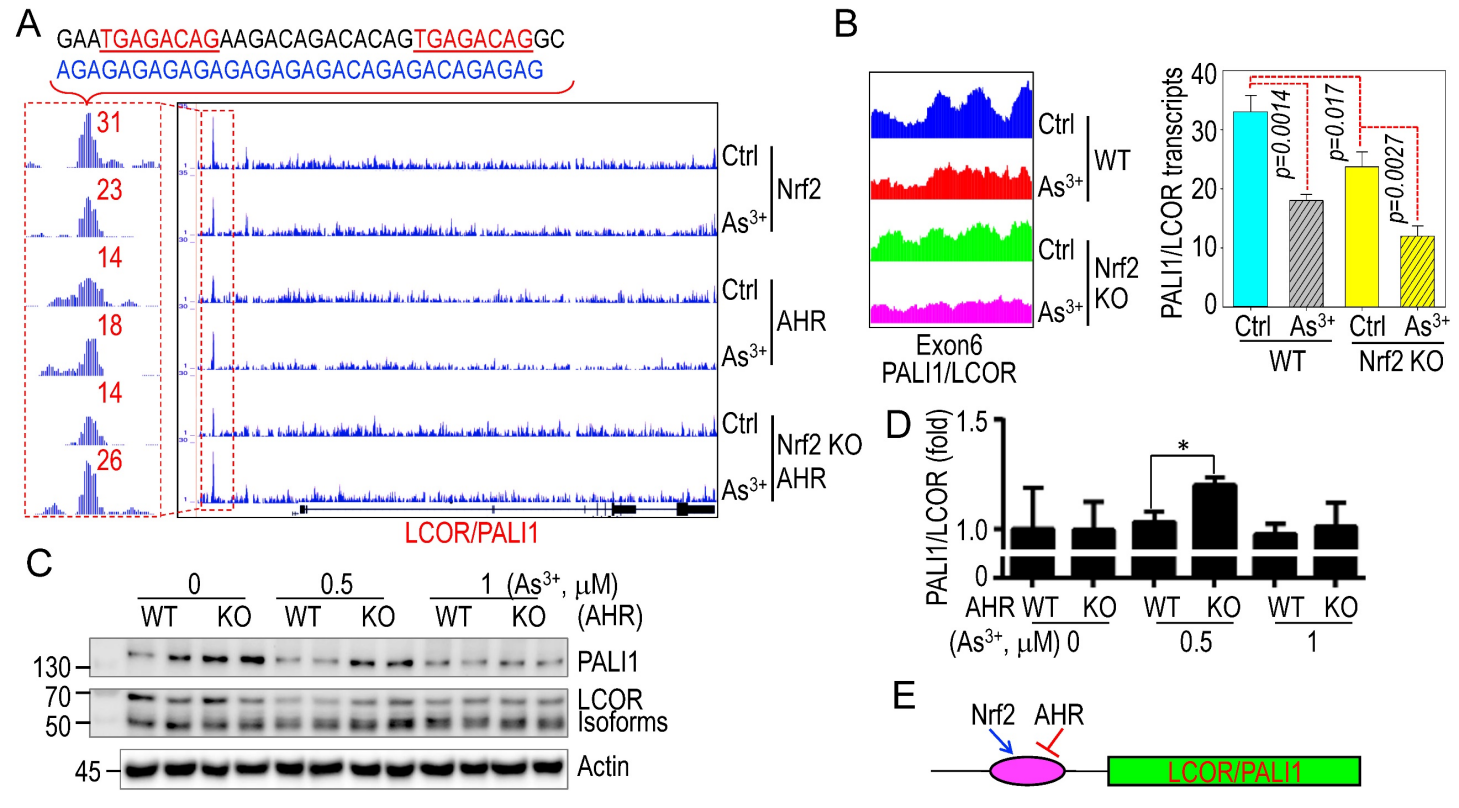
**Opposing regulation of Nrf2 and AHR on PALI1 expression in response to As^3+^. A** ChIP-seq analysis reveals enrichment of Nrf2 and AHR binding at a region 30.7 kb upstream of the PALI1 gene in both control and As^3+^-treated cells. Notably, AHR binding is enhanced in Nrf2 KO cells treated with As^3+^. The DNA sequence above the panel highlights a non-canonical Nrf2 binding elements and an AGAG/ACAG-rich region corresponding to the Nrf2/AHR binding peaks. **B** RNA-seq profiles and semi-quantitative analysis of PALI1 mRNA levels in WT and Nrf2 KO cells under control and As^3+^ treatment conditions. Data are expressed as mean ± SD, n = 3. **C** Western blot analysis of PALI1 and LCOR expression in WT and AHR KO cells, with or without As^3+^ treatment. **D** qPCR analysis showing the relative ratios of PALI1 and LCOR mRNA in WT and AHR KO cells, with or without As^3+^ treatment. Data are expressed as mean ± SD, n = 6, *p <0.05 vs. the corresponding control. **E** A simplified diagram illustrating that Nrf2 promotes, while AHR suppresses, PALI1 expression.

Previous studies demonstrated that As^3+^ strongly activates the oncogenic transcription factor Nrf2 while inhibiting the tumor suppressor-like xenobiotic transcription factor AHR 30, 33, 41. In ChIP-seq analysis, we noted overlapping enrichment peaks of Nrf2 and AHR in a 30.7 kb upstream AGAG/ACAG-rich region of the LCOR/PALI1 gene locus (**Fig. 5A**). Two non-canonical Nrf2 binding motifs (TGAGACAG), differing by a single nucleotide from the canonical Nrf2 binding sequences (TGAGTCAG or TGACTCAG), were identified at the summit of the Nrf2 ChIP-seq peak. These sites were immediately followed by an approximately 140 bp region enriched in AGAG/ACAG repeat sequences. Upon As³⁺ treatment, Nrf2 occupancy at this locus was markedly reduced, whereas AHR binding was enhanced. Notably, AHR enrichment was further increased in CRISPR-mediated Nrf2 knockout (KO) BEAS-2B cells, suggesting a compensatory or competitive regulatory mechanism between Nrf2 and AHR at this locus (**Fig. 5A**). RNA-seq data showed that Nrf2 knockout reduced basal PALI1 expression, which was further decreased by As^3+^ treatment (**Fig. 5B**). Conversely, PALI1 protein levels, but not LCOR isoforms, were elevated in AHR KO cells (**Fig. 5C**), a result further supported by qPCR showing increased PALI1 mRNA levels in AHR KO cells treated with As^3+^ (**Fig. 5D**). These findings implicate that Nrf2 and AHR play opposite roles in mediating As^3+^-induced suppression of PALI1 (**Fig. 5E**).

### 3.6. PALI1 as a potential tumor suppressor in multiple cancers

**Figure 6:**
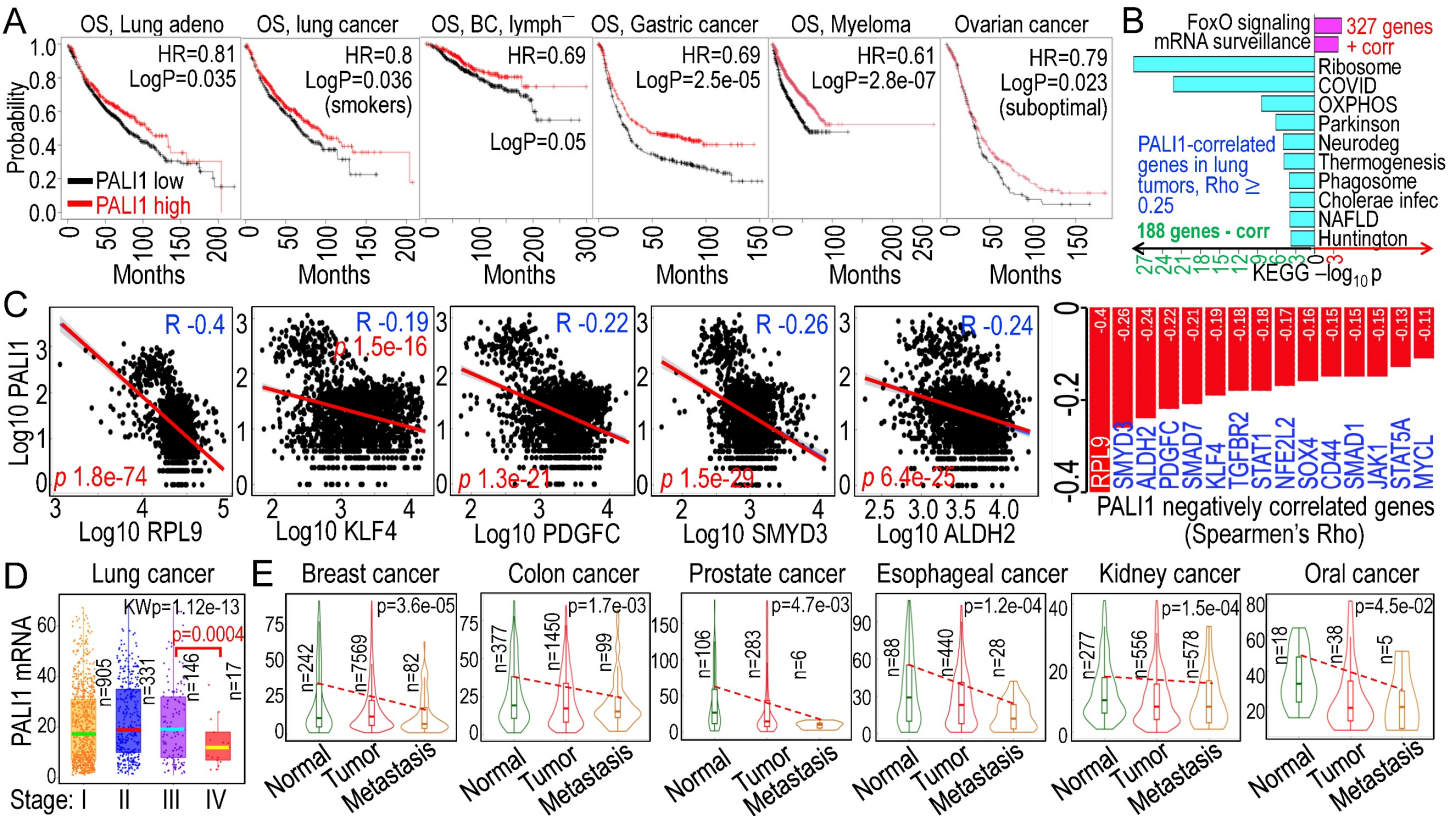
**Tumor suppressor-like properties of PALI1 in human cancers. A** Higher PALI1 expression levels are associated with improved overall survival (OS) in patients with the indicated cancer. **B** Functional pathways of the genes positively and negatively correlated with PALI1 in human lung cancer. **C** Negative correlation of PALI1 with the indicated genes associated with cancer stem-like cells (CSCs). **D** PALI1 expression status in different stages of lung cancer, which showed significantly reduced PALI1 expression in stage IV lung cancer. **E** Reduced PALI1 expression in primary tumor tissues, with further decreases observed in metastatic tumors of the indicated cancer types.

While PALI1 expression is elevated in some prostate tumors 35, its oncogenic or tumor-suppressive function may be context-dependent. Higher PALI1 expression (using C10orf12 as a surrogate in database searches) correlates with improved overall survival (OS) in lung adenocarcinoma, smoke-related lung cancer, breast cancer without lymph node metastasis, gastric cancer, myeloma, and suboptimal ovarian cancer (**Fig. 6A**). To gain insight into how higher PALI1 expression predicts better survival in cancer patients, we examined genes correlated with PALI1 in human lung cancer and found 327 genes positively correlated and 188 genes negatively correlated with PALI1, with a Spearman correlation coefficient (Rho) ≥ 0.25. Pathway analysis revealed that the positively correlated genes are mainly associated with FoxO signaling and mRNA surveillance, while the negatively correlated genes are enriched in pathways related to ribosomal biogenesis, oxidative phosphorylation (OXPHOS) in mitochondria, and inflammation (**Fig. 6B**), indicating that PALI1 is more likely a negative regulator for the pro-oncogenic signaling. Indeed, several genes known to be important in the stemness of the cancer cells, including RPL9 42, 43, KLF4 44, 45, PDGFC 46, 47, SMYD3 48, 49, and ALDH2 50, 51, are negatively correlated with PALI1 expression, suggesting that PALI1 may function as an antagonist to stemness-associated transcriptional programs (**Fig. 6C**). In human lung cancer tissues, PALI1 mRNA levels remained relatively stable from stage I to III, but showed a marked reduction in stage IV, indicating a potential link between PALI1 downregulation and cancer progression, such as metastasis (**Fig. 6D**). Moreover, across multiple cancer types, including breast, colon, prostate, esophageal, kidney, and oral cavity cancer, PALI1 expression is consistently decreased in primary tumor, with a further reduction observed in metastatic lesions (**Fig. 6E**). These findings support a tumor suppressor-like role for PALI1 and highlight that As³⁺-mediated repression of PALI1 may be a key mechanism by which As^3+^ promotes carcinogenesis through disruption of chromatin-based tumor suppressive pathways.

## 4. Discussion

Contamination of food and water supplies with As^3+^ continues to be a critical and widespread global public health concern, affecting an estimated 200 million individuals across various geographic regions. Chronic exposure to As^3+^ has been epidemiologically linked to an increased risk of multiple malignancies, including cancers of the lung, skin, bladder, liver, kidney, and prostate 1, 7, 9. The molecular mechanisms underlying As^3+^-induced carcinogenesis are complex and not yet fully understood. Multiple pathways have been implicated, including DNA repair disruption, aberrant cellular signaling, and epigenetic reprogramming 29, 52, 53. In the present study, we provide novel evidence demonstrating that As^3+^ exerts a potent repressive effect on the expression of PALI1, a protein involved in the regulation of chromatin architecture and transcriptional repression 35, 39, 54, 55. This repression of PALI1 subsequently impairs the function of PRC2.1, a crucial variant of the PRC2, which is responsible for the deposition and maintenance of H3K9me3 and H3K27me3 markers at specific regions or gene loci. H3K9me3, a histone modification associated with constitutive heterochromatin, plays a key role in maintaining genome stability and regulating the expression of transposable elements. The loss of H3K9me3 results in the de-repression of transposable elements, leading to genomic instability, altered gene expression, and disruption of cellular homeostasis, features widely recognized as hallmarks of cancer.

A number of methyltransferases and demethylases are involved in the regulation of H3K9me3 across the genome 56, 57. These enzymes typically form complexes with regulatory subunits and accessory proteins. Emerging evidence suggests that, unlike the PRC2.2 variant, which catalyzes H3K27me3 solely through the core subunit EZH2, PRC2.1 can catalyze both H3K9me3 via PALI1-G9a and H3K27me3 through EZH2 35, 39, 58. Under normal conditions, PRC2.1 and PRC2.2 exist in equilibrium, maintained by the balanced availability of their accessory proteins: PALI1 for PRC2.1 and JARID2 for PRC2.2. Both PALI1 and JARID2 compete for binding to the SUZ12 subunit (**Fig. 3A**), so limiting the availability of either protein shifts the balance between PRC2.1 and PRC2.2. In our analysis, we found that the effect of As^3+^ on the enzymes responsible for H3K9me3, including its methyltransferases and demethylases (data not shown) is marginal. Instead, transcriptomic and protein analyses showed that As^3+^ significantly downregulates PALI1, thereby limiting PRC2.1 activity on H3K9me3 and favoring PRC2.2 activity. This selective regulation of PRC2.1 and PRC2.2, driven by PALI1 suppression, accounts the discordant effects of As^3+^ on H3K9me3 and H3K27me3 levels. Given that H3K9me3 is crucial for maintaining genomic stability, regulating gene expression dynamics, and preserving cell identity 56, 59, 60, PALI1 inhibition will weaken the overall tumor suppressive potential.

While our RNA-seq data (**Figure 3B**) show that many PRC2 components—including PALI1, PALI2, MTF2, and others—are transcriptionally downregulated following acute As³⁺ exposure, we observed a paradoxical increase in global H3K27me3 levels. This may be explained by several possibilities: Firstly, despite reduced mRNA levels of some PRC2 components, the protein levels of core subunits EZH2 and SUZ12 remain largely unchanged in our Western blot analyses. Thus, sufficient PRC2 enzymatic activity may be retained to drive an increased H3K27me3 deposition. Secondly, As³⁺ exposure may modify chromatin structure or histone reader/writer dynamics in a way that enhances PRC2 recruitment or activity at specific loci, thereby promoting H3K27me3 accumulation independently of subunit abundance. Furthermore, As³⁺ has been reported to disrupt the function of certain histone demethylases, such as KDM6A/B (UTX/JMJD3), which actively remove H3K27me3 marks. A reduction in demethylase activity could lead to a net increase in H3K27me3 even in the presence of lower PRC2 transcription. Given that H3K9me3 is crucial for maintaining genomic stability, regulating gene expression dynamics, and preserving cell identity 55, 58, 59, PALI1 inhibition may represent an underappreciated mechanism through which As³⁺ contributes to malignant transformation and carcinogenesis in humans.

In previous studies, we reported that As^3+^ enhances Nrf2 binding while reducing AHR binding to genes involved in energy metabolism, oncogenesis, and stemness in cancer cells 30, 33, 41. Intriguingly, at the *PALI1* locus, As³⁺ induces the opposite pattern: decreased Nrf2 and increased AHR binding upstream of the gene. Unlike other oncogenic targets of Nrf2, this region lacks a canonical antioxidant response element (ARE) and instead contains non-canonical Nrf2 motifs and a 140 bp AGAG/ACAG-enriched repeat sequence. ChIP-seq analysis of histone methylation revealed distinct, sharp peaks of H3K9me3 and H3K27me3, two well-known repressive markers involved in heterochromatin formation, coinciding with the Nrf2 peak. These repressive marks, recruited by the repetitive elements, may therefore override the transcriptional activation normally mediated by Nrf2. The exact contribution of these repeat elements to the reversal of Nrf2/AHR dynamics at the PALI1 gene remains unclear but may be central to its silencing by As³⁺. Beyond transcriptional repression, other post-transcriptional mechanisms may contribute to PALI1 downregulation. For example, As³⁺ has been shown in other systems to influence mRNA stability via modulation of RNA-binding proteins or non-coding RNAs 61. Whether such mechanisms apply to *PALI1* remains unknown but is a promising area for future investigation. Moreover, we cannot exclude the possibility that As³⁺ promotes PALI1 protein degradation through the ubiquitin-proteasome system, a well-documented cellular response to toxic stress 62. Further studies using inhibitors of transcription, translation, and proteasomal degradation, as well as RNA decay assays, will be needed to dissect these potential mechanisms.

Importantly, H3K9me3 is a key repressive mark involved in the silencing of the LINE-1 (L1) family of retrotransposons, which constitute approximately 17% of the human genome 63, 64. Although the majority of LINE-1 elements are truncated and inactive, a subset of 80-100 full-length LINE-1s retains mobilization potential via their encoded proteins, ORF1p and ORF2p 63. Activation of these retrotransposons can result in insertional mutagenesis, chromosomal rearrangements, and DNA double-strand breaks, potent sources of genomic instability. Our findings suggest that As³⁺-mediated downregulation of PALI1, through reduction of PRC2.1 activity and subsequent H3K9me3 loss, may lead to the reactivation of LINE-1 elements and contribute to genome destabilization. This expands the oncogenic potential of As³⁺ beyond its known effects on cellular signaling and metabolic pathways, implicating transposable element dysregulation as a critical component of As^3+^-induced carcinogenesis.

The first evidence implicating PALI1 in PRC2 function was reported by Smits et al and Alekseyenko et al, who independently demonstrated a physical interaction between C10orf12 and PRC2 using cross-linking and affinity purification in HeLa and 293 cells, respectively 40, 65. At the time, however, it was not recognized that C10orf12 was part of an alternatively spliced transcript of LCOR until the findings of Hauri et al, who identified that it is not only a facultative subunit of PRC2.1 but also a product of LCOR alternative splicing 66. Subsequent studies confirmed the existence of PRC2 associated LCOR isoform 1 (PALI1), which arises from alternative splicing of LCOR 39, 54, 55, 58. Additional evidence has highlighted critical roles for PALI1 and its associated PRC2.1 in embryonic development and human prostate cancer 35, 67. In the present report, we identify PALI1 as a potential tumor suppressor and uncover a previously unrecognized mechanism by which As³⁺ promotes genomic instability and malignant transformation through PALI1 repression. This repression disrupts PRC2.1-mediated H3K9me3 deposition, leading to reactivation of LINE-1 elements. Given the essential role of LINE-1 silencing in maintaining genome integrity, further investigation into the interplay between PALI1, H3K9me3, and transposable element regulation may yield new insights into As³⁺-induced epigenetic toxicity and cancer risk.

## Abbreviations

As^3+^: Inorganic arsenic
BER: Base excision repair
ChIP-seq: Chromatin immunoprecipitation and sequencing
Co-IP: Co-immunoprecipitation
CSCs: Cancer stem-like cells
GPCR: G-protein-coupled receptor
H3K27me3: Histone H3 lysine 27 trimethylation
H3K4me3: Histone H3 lysine 4 trimethylation
H3K9me3: Histone H3 lysine 9 trimethylation
LINE-1: long interspersed nuclear elements-1
MMP: Matrix metalloproteinase
NER: Nucleotide excision repair
OS: Overall survival
OXPHOS: Oxidative phosphorylation
PALI1: PRC2-associated LCOR isoform 1
PRC2.1: Polycomb repressive complex 2.1
ROS: Reactive oxygen species
